# *Glaesserella parasuis* infection triggers endoplasmic reticulum stress-mediated pyroptosis via PERK/eIF2α/ATF4 axis and metabolic reprogramming in porcine alveolar macrophages

**DOI:** 10.1186/s13567-025-01580-2

**Published:** 2025-07-15

**Authors:** Weili Feng, Yu Han, Wanyun Zhang, Lei Wu, Ao Zhou, Liangyu Shi, Jing Zhang

**Affiliations:** Laboratory of Genetic Breeding, Reproduction and Precision Livestock Farming & Hubei Provincial Center of Technology Innovation for Domestic Animal Breeding, School of Animal Science and Nutritional Engineering, Wuhan Polytechnic University, Wuhan, 430023 China

**Keywords:** *Glaesserella parasuis*, porcine alveolar macrophages, apoptosis, pyroptosis, endoplasmic reticulum stress, metabolic reprogramming

## Abstract

*Glaesserella parasuis*, the causative agent of Glässer’s disease in swine, triggers severe systemic inflammation; however, the molecular mechanisms underpinning its pathogenesis remain incompletely understood. This study investigated the cellular and metabolic responses of porcine alveolar macrophage 3D4/21 cells to *G. parasuis* infection. Exposure to the pathogen significantly reduced cell viability and up-regulated pro-inflammatory cytokines (IL-6, IL-8, IL-1β, and TNF-α). Mechanistically, *G. parasuis-*induced apoptosis via up-regulation of Bcl-2-associated X protein (Bax) and cleaved Caspase-3, coupled with down-regulation of B-cell lymphoma-2 (Bcl-2). By contrast, pyroptosis was characterised by activation of the NOD-like receptor family pyrin domain containing 3 (NLRP3) inflammasome, Caspase-1 cleavage, and gasdermin D (GSDMD)-mediated membrane pore formation. Notably, infection provoked endoplasmic reticulum (ER) stress through the PERK/eIF2α/ATF4/CHOP pathway, as evidenced by ER expansion, ribosomal detachment, and mitochondrial damage. Treatment with the ER stress inhibitor 4-phenylbutyric acid (4-PBA) mitigated these alterations. Inhibition of PERK with GSK2656157 suppressed pyroptosis-related proteins (NLRP3, GSDMD, and Caspase-1) without altering apoptosis markers, indicating the existence of distinct regulatory pathways. Untargeted metabolomic profiling revealed extensive metabolic reprogramming, identifying 419 differentially expressed metabolites associated with pathways such as glutathione metabolism, glycerophospholipid metabolism, and arachidonic acid metabolism, underscoring their involvement in *G. parasuis* infection and immune modulation. Collectively, these findings demonstrate that *G. parasuis* undermines host defences by activating PERK-mediated ER stress to drive pyroptosis, while simultaneously inducing apoptosis and metabolic dysregulation through independent mechanisms. This study provides novel insights into *G. parasuis* pathogenesis and highlights the PERK pathway and metabolic regulators as potential therapeutic targets for mitigating Glässer’s disease.

**Supplementary Information:**

The online version contains supplementary material available at 10.1186/s13567-025-01580-2.

## Introduction

*Glaesserella parasuis* (*G. parasuis*, GPS; previously *Haemophilus parasuis*), a Gram-negative bacterium belonging to the *Pasteurellaceae* family, is the causative agent of Glässer’s disease in pigs. This systemic infectious disease is characterised by polyserositis, arthritis, and meningitis, predominantly affecting weaned piglets and resulting in substantial economic losses within the swine industry [1].

Infection with *G. parasuis* elicits a pronounced inflammatory response, characterised by elevated levels of IL-1β, IL-6, TNF-α, and IL-17, which collectively contribute to tissue damage and systemic inflammation [2, 3]. Recent studies have demonstrated that infection with *G. parasuis* serotype 5 triggers an inflammatory response by activating the NLRP3 inflammasome [4] and induces pyroptosis through the cleavage of gasdermin D (GSDMD) and the activation of Caspase-1, thereby amplifying the inflammatory cascade [5]. Moreover, Li et al. reported that *G. parasuis* induces cell cycle arrest and promotes apoptosis in a p53-dependent manner [6]. While these studies highlight the key inflammatory and cell death pathways activated by *G. parasuis*, the upstream events that precipitate these responses, particularly those involving organelle stress and metabolic reprogramming, remain poorly elucidated.

Emerging evidence indicates that endoplasmic reticulum (ER) stress plays a pivotal role in modulating host–pathogen interactions. Microbial infections frequently disrupt ER homeostasis, thereby activating the unfolded protein response (UPR). This response is mediated via three principal sensors: inositol-requiring transmembrane kinase endoribonuclease-1α (IRE1α), protein kinase RNA-like ER Kinase (PERK), and activating transcription factor 6 (ATF6) [7–9]. This evolutionarily conserved stress response regulates protein folding and also orchestrates inflammatory signalling and cell fate determination. For instance, *Mycobacterium tuberculosis* infects macrophages and triggers apoptosis through ER stress-mediated pathways [10]. Nie et al. further demonstrated that *Bacillus Calmette-Guérin* infection mediates NLRP3 inflammasome activation and pyroptosis in THP-1 macrophages via ER stress [11]. However, the interplay between ER stress and *G. parasuis* infection in porcine alveolar macrophages (PAMs), the primary cellular niche for this pathogen, remains unexplored.

Concomitant with stress signalling, metabolic reprogramming represents a hallmark of macrophage-pathogen encounters. Bacterial infections can frequently disrupt host cell metabolism, playing a crucial role in determining the outcome of infection [12]. Previous studies have demonstrated that bacterial infections can reprogramme host cell metabolism, resulting in changes in glycolysis, amino acid metabolism, and lipid metabolism [13, 14]. Guimarães et al. identified the pivotal role of the UPR in regulating macrophage metabolic function during *Brucella abortus* infection [15]. However, the specific metabolic adaptations induced by *G. parasuis* in PAMs remain poorly defined.

Here, we employed the 3D4/21 PAM cell line to systematically analyse the multidimensional host responses to *G. parasuis* infection. By integrating analyses of cellular viability, inflammatory signalling, apoptotic and pyroptotic pathways, ER stress dynamics, and metabolic alterations, this study aims to establish the functional links between ER stress and inflammatory cell death during infection, while also delineating pathogen-induced metabolic adaptations in PAMs. Collectively, these findings advance our understanding of *G. parasuis* pathogenesis and highlight potential targets for therapeutic intervention and disease control.

## Materials and methods

### Bacterial strain

The highly virulent *G. parasuis* serovar 5 strain SH0165 used in this study was kindly provided by Dr Shulin Fu (Hubei Key Laboratory of Animal Nutrition and Feed Science, Wuhan Polytechnic University, China) [16]. The bacteria were cultured at 37 °C in Trypticase Soy Broth (Difco Laboratories, USA) supplemented with 0.01% nicotinamide adenine dinucleotide (Sigma, USA) and 10% foetal bovine serum (MCE, USA).

### Cell culture and bacterial infection

The porcine alveolar macrophage cell line 3D4/21 was cultured in RPMI 1640 medium (HyClone, USA) supplemented with 10% foetal bovine serum (MCE, USA) and 1% penicillin–streptomycin solution (Beyotime, China), and maintained at 37 °C in a humidified atmosphere containing 5% CO₂.

The 3D4/21 cells were seeded into appropriate culture plates and infected with *G. parasuis* at a multiplicity of infection (MOI) of 10 or 100, corresponding to an average of 10 or 100 bacterial cells per host cell. The cells were then incubated for 24 or 48 hours. For co-treatment experiments, cells were seeded into 6-well plates at a density of 2 × 10^5^ cells/well and allowed to adhere overnight. They were then pre-treated for 2 h with either 4-phenylbutyric acid (4-PBA; 5 mM), an ER stress inhibitor, or GSK2656157 (10 µM), a selective PERK inhibitor, followed by infection with *G. parasuis* at an MOI of 10. The cells were subsequently cultured for an additional 24 h before sample collection and subsequent analysis. Both compounds were obtained from MedChemExpress (MCE, USA).

### Scanning electron microscopy analysis

Scanning electron microscopy (SEM) analysis was performed on two groups: an uninfected control and an infected group (MOI of 10 for 24 h), with three biological replicates (*n* = 3). The 3D4/21 cells (2 × 10^5^ cells/well) were seeded onto sterile coverslips in a 6-well plate and infected with *G. parasuis* at an MOI of 10, or left uninfected. After 24 h, the culture medium was removed and the samples were gently washed with phosphate-buffered saline (PBS; HyClone, USA) before being fixed with electron microscopy fixative (Servicebio, China).

Post-fixation, samples were washed with 0.1 M phosphate buffer (PB, pH 7.4; Servicebio, China), incubated in 1% osmium tetroxide (OsO_4_; Ted Pella, USA) for 2 h, then dehydrated through a graded ethanol series (Sinopharm, China) and treated with isoamyl acetate (Sinopharm, China) for 15 min. Samples were subsequently dried using a critical point dryer (Quorum Technologies Ltd., UK). For conductive coating, specimens were mounted on metallic stubs with carbon stickers and sputter-coated with gold for 30 s. Imaging was performed using a scanning electron microscope (Hitachi, Japan).

### Cell viability assay

Cell viability was assessed using the CellTiter-Lumi™ Plus Luminescent Cell Viability Assay kit (Beyotime, China). Briefly, 3D4/21 cells were seeded into a 24-well plate at a density of 1 × 10^5^ cells per well and incubated overnight. The cells were then infected with *G. parasuis* at an MOI of 10 or 100 and incubated for 24 or 48 h. Each condition was performed in triplicate. Following infection, cell viability was assessed according to the manufacturer’s instructions. The CellTiter-Lumi^™^ Plus reagent was added to each well, and the plate was gently shaken at room temperature for 2 min to ensure complete lysis. After a further 10 min of incubation at room temperature, luminescence signals were measured using a microplate reader (BIO-RAD, USA).

### RNA isolation and quantitative real-time PCR (qRT-PCR)

To assess the expression of pro-inflammatory cytokine genes in 3D4/21 cells, total RNA was extracted after infection with *G. parasuis* at an MOI of 10 or 100 for 24 or 48 h. Each condition was performed in triplicate.

Total RNA was extracted using TRIzol reagent (Invitrogen, USA) according to the manufacturer’s protocol. Single-stranded cDNA was synthesised using the Evo M-MLV RT Mix kit with gDNA Clean for quantitative polymerase chain reaction (qPCR) (Accurate Biotechnology, China). Quantitative reverse transcription polymerase chain reaction (qRT-PCR) was performed with the SYBR Green Premix Pro Taq HS qPCR kit II (Accurate Biotechnology, China) on a QuantStudio 1 Plus real-time PCR system (Applied Biosystems, USA), according to the manufacturer’s instructions. *GAPDH* was used as the internal reference gene. The relative mRNA expression levels of *IL-1β*, *IL-6*, *IL-8*, and *TNF-α* were analysed using the 2^−ΔΔCT^ method. Primer sequences are listed in Additional file 1.

### Annexin V-FITC apoptosis assay

Apoptosis was assessed using the Annexin V-FITC Apoptosis Detection kit (Beyotime, China). Briefly, 3D4/21 cells were either infected with *G. parasuis* (MOI = 10) for 24 h or left uninfected and then harvested for analysis. Each group (control and infected) comprised three biological replicates. Cells were trypsinised (0.25%) and centrifuged to remove the supernatant. Following three washes with cold PBS, the cells were resuspended in 195 μL of binding buffer provided by the kit. The cells were incubated with Annexin V-FITC (5 μL) and propidium iodide (PI; 10 μL) at room temperature in the dark for 20 min. Flow cytometry analysis (Beckman, USA) was performed to detect and quantify apoptosis.

### Western blot analysis

Total cellular protein was extracted using RIPA lysis buffer (R0278, Sigma-Aldrich, USA) supplemented with 2% protease–phosphatase inhibitor (P1050, Beyotime, China). Protein concentration was determined with a bicinchoninic acid (BCA) protein assay kit (P0010, Beyotime, China). Equal amounts of protein (20 μg per sample) were loaded onto a polyacrylamide gel for electrophoresis. Following electrophoresis, proteins were transferred to polyvinylidene difluoride (PVDF) membranes (Immobilon-P, Merck Millipore, USA). The membranes were blocked with Tris-buffered saline with Tween 20 (TBST) containing 5% skim milk (GC310001, Servicebio, China) for 2 h at room temperature.

Subsequently, the membranes were incubated overnight at 4 °C with the following primary antibodies: cleaved-caspase 3 (WL01992, Wanleibio, China), Bax (WL01637, Wanleibio, China), Bcl-2 (WL01556, Wanleibio, China), CHOP (WL00880, Wanleibio, China), GRP78 (WL03157, Wanleibio, China), ATF4 (WL02330, Wanleibio, China), eIF2α (WL01909, Wanleibio, China), p-eIF2α (310073, Zenbio, China), JNK (WL01295, Wanleibio, China), p-JNK (WL01813, Wanleibio, China), IRE1 (WL02562, Wanleibio, China), p-IRE1 (WL05299, Wanleibio, China), PERK (WL03378, Wanleibio, China), p-PERK (WL05295, Wanleibio, China), NLRP3 (WL02635, Wanleibio, China), GSDMD (WL05686, Wanleibio, China), Caspase-1 (WL02996, Wanleibio, China), and GAPDH (AC001, ABclonal, China). After washing, membranes were incubated with an HRP-conjugated goat anti-rabbit IgG secondary antibody (WLA023, Wanleibio, China) for 1 h at room temperature. Protein bands were visualised with BeyoECL Star (P0018AS, Beyotime, China) following the manufacturer’s instructions. Western blot results were quantified using ImageJ software, with GAPDH serving as the internal reference. Protein experiments were performed with four biological replicates per condition.

### Transmission electron microscope (TEM) analysis

Four treatment groups were established, each with three biological replicates: (1) a non-infected control group, (2) a *G. parasuis* infection group (MOI = 10), (3) a group treated with 5 mM 4-PBA alone, and (4) a group pre-treated with 5 mM 4-PBA for 2 h, followed by *G. parasuis* infection. After 24 h, the cells were collected through centrifugation, resuspended in electron microscopy fixative (Servicebio, China), and stored at 4 °C.

For pre-embedding, the fixed samples were washed three times with 0.1 M phosphate buffer (PB; pH 7.4; Servicebio, China) and embedded in 1% agarose (Sinopharm, China). Post-fixation was performed in 1% OsO_4_ (Ted Pella, USA) for 2 h at room temperature, followed by three washes with 0.1 M PB (Servicebio, China). Samples were dehydrated through a graded ethanol and acetone series (Sinopharm, China), then infiltrated and embedded in resin. Ultrathin Sects. (80 nm) were cut using an ultramicrotome (Leica Microsystems, Germany), mounted on 150-mesh copper grids, and subsequently stained with 2% uranyl acetate (SPI Supplies, USA) and 2.6% lead citrate (Sigma, USA). Imaging was carried out using a transmission electron microscope (Hitachi, Japan).

### Untargeted metabolomics analysis

Untargeted metabolomic profiling was performed to assess metabolic changes in 3D4/21 cells following infection with *G. parasuis*. Cells were treated with or without *G. parasuis* (MOI = 10) for 24 h, with six biological replicates per group. Following treatment, the cells were harvested and stored at −80 °C.

For extraction, samples were thawed on ice. A total of 500 μL of a methanol–water mixture (4:1, v/v) containing an internal standard was then added to each tube, followed by 3 min of vortexing. Samples subsequently underwent three freeze–thaw cycles, each comprising 5 min in liquid nitrogen, 5 min on dry ice, thawing on ice, and 2 min of vortexing per cycle. Following centrifugation at 12 000 rpm for 10 min at 4 °C, 300 μL of the supernatant was collected, incubated at –20 °C for 30 min, and then centrifuged again at 12 000 rpm for 3 min at 4 °C. A 200 μL aliquot of the resulting supernatant was used for metabolomics analysis.

Metabolite detection was conducted by MetWare Biotechnology Co., Ltd. (Wuhan, China). Metabolites were determined via ultra-performance liquid chromatography-tandem mass spectrometry (UPLC–MS/MS). Instrumental analysis was performed on the UPLC system (Shimadzu Nexera UHPLC LC-30A) coupled to a Sciex TripleTOF 6600 mass spectrometer (Q-TOF, AB Sciex, Toronto, Canada). Chromatographic separation was achieved on a Waters ACQUITY Premier HSS T3 column (1.8 µm, 2.1 mm × 100 mm). The mass spectrometer operated in information-dependent acquisition mode. Analytical conditions are provided in Additional file 2.

Raw LC–MS data were converted to mzML format using ProteoWizard. Peak extraction, alignment, and retention time correction were performed using the XCMS software (version 3.7.1). Peak area normalisation was conducted using the support vector regression (SVR) method. Metabolites detected in fewer than 50% of samples in any group were excluded from further analysis. Compound identification was performed using MetWare Biotechnology Co., Ltd.’s in-house high-resolution MS/MS database, together with several integrated public and predictive databases, including METLIN [17], HMDB 4.0 [18], KEGG [19], MoNA [20], MassBank [21], and metDNA [22]. Additionally, compound annotation was supported by an AI-based predictive spectral library generated with the CFM-ID algorithm [23].

Orthogonal partial least squares-discriminant analysis (OPLS–DA) was applied to evaluate the identified metabolites. Differential metabolites between groups were selected based on variable importance in projection (VIP > 1) and statistical significance (*P* < 0.05). VIP values were obtained from the OPLS–DA model, with score and permutation plots generated using the R package MetaboAnalystR. Data were pre-processed by log_2_ and mean centring prior to analysis. A permutation test (200 iterations) was conducted to validate the model and prevent overfitting.

Metabolites were annotated using the KEGG Compound database and mapped to the KEGG Pathway database for pathway enrichment analysis.

### Statistical analysis

All results are presented as mean ± standard deviation (SD). Statistical analysis was conducted using unpaired Student’s *t* tests for two-group comparisons and one-way ANOVA with a Bonferroni test for multiple-group comparisons. All analyses were performed in GraphPad Prism 8. Significant differences were indicated as follows: *p < 0.05, **p < 0.01, and ***p < 0.001; ns indicates not significant.

## Results

### Effects of *G. parasuis* infection on cell viability and inflammation in 3D4/21 cells

**Figure 1:**
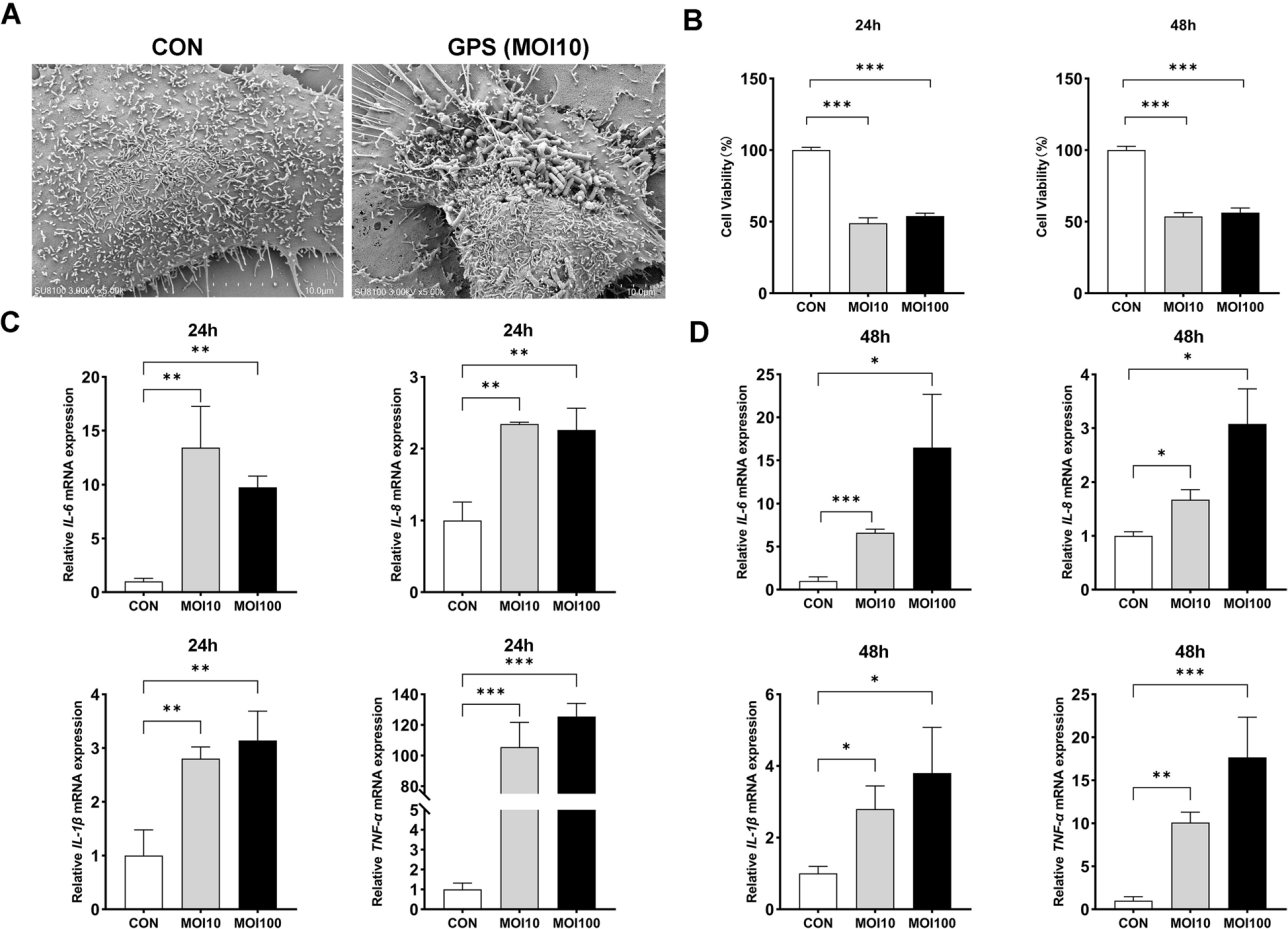
***G. parasuis*** **infection reduces cell viability and triggers inflammatory responses in 3D4/21 cells**. **A** Scanning electron microscopy images of 3D4/21 cells with or without *G. parasuis* infection (magnification: × 5.0 k). **B** Cell viability assessed by the CellTiter-Lumi™ Plus assay at 24 and 48 hpi under different MOIs. **C**, **D** Relative mRNA expression levels of inflammatory cytokines (IL-6, IL-8, IL-1β, and TNF-α) measured at 24 and 48 hpi. Data are presented as mean ± SD (*n* = 3), with significance compared to the control group (CON, uninfected cells) indicated as *p < 0.05, **p < 0.01, ***p < 0.001.

SEM was used to observe bacterial adherence on 3D4/21 cells following infection with *G. parasuis*. After 24 h of exposure at an MOI of 10, bacterial adherence was clearly visible on the surface of infected cells, whereas no such attachment was observed in control cells (Figure 1A).

To investigate the effects of *G. parasuis* infection on cell viability and inflammatory responses, 3D4/21 cells were infected at MOIs of 10 and 100 for 24 and 48 h. Cell viability assays revealed a significant reduction in viability under both infection conditions compared to uninfected controls (*p* < 0.05; Figure 1B). Additionally, RT-qPCR analysis showed that the mRNA levels of IL-6, IL-8, IL-1β, and TNF-α were significantly increased following *G. parasuis* infection (*p* < 0.05; Figures 1C and D). At 24 h, both MOI 10 and MOI 100 markedly up-regulated all four cytokines, with TNF-α showing an approximately 100-fold increase. At 48 h, IL-6, IL-8, and IL-1β levels further increased under both MOI conditions, while TNF-α remained elevated but with a reduced fold change (~ tenfold), indicating that *G. parasuis* infection induces a robust inflammatory response.

### Effects of *G. parasuis* infection on apoptosis in 3D4/21 cells

**Figure 2:**
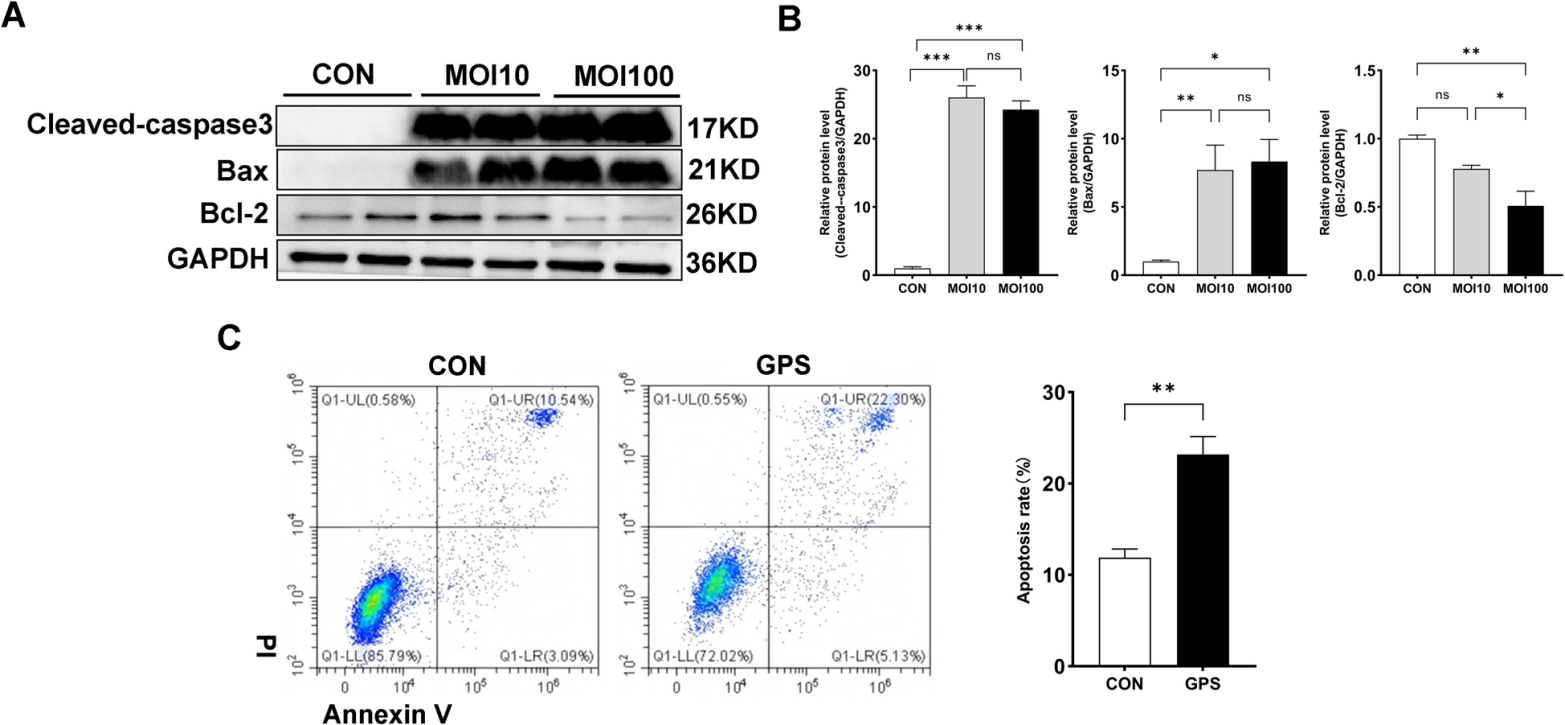
***G. parasuis*** **infection induces apoptosis in 3D4/21 cells.** **A** Western blot analysis of Cleaved Caspase-3, Bax, and Bcl-2 protein expression. **B** Quantification of protein expression levels using ImageJ. Data are presented as mean ± SD (*n* = 4). **C** Detection of cell apoptosis in 3D4/21 cells using flow cytometry after treatment with *G. parasuis* (MOI 10) for 24 h. Data are presented as mean ± SD (*n* = 3). Significance relative to the control group: *p < 0.05, **p < 0.01, ***p < 0.001. Ns indicates no significant difference. CON indicates the uninfected control group, and GPS indicates the *G. parasuis*-infected group.

To assess the impact of *G. parasuis* infection on apoptosis, 3D4/21 cells were exposed to the pathogen at MOIs of 10 and 100 for 24 h. Western blot analysis revealed a significant increase in the expression of cleaved caspase-3 and Bax at both MOIs (*p* < 0.05), along with a notable decrease in Bcl-2 expression at an MOI of 100 compared to the control group (*p* < 0.05; Figures 2A and B).

Apoptosis was further evaluated using Annexin V/PI staining followed by flow cytometry. Infection *with G. parasuis* for 24 h significantly increased the percentage of apoptotic cells and the overall apoptosis rate compared to controls (Figure 2C). Together, these findings demonstrate that *G. parasuis* infection induces significant apoptosis in 3D4/21 cells.

### Effects of *G. parasuis* infection on pyroptosis in 3D4/21 cells

**Figure 3:**
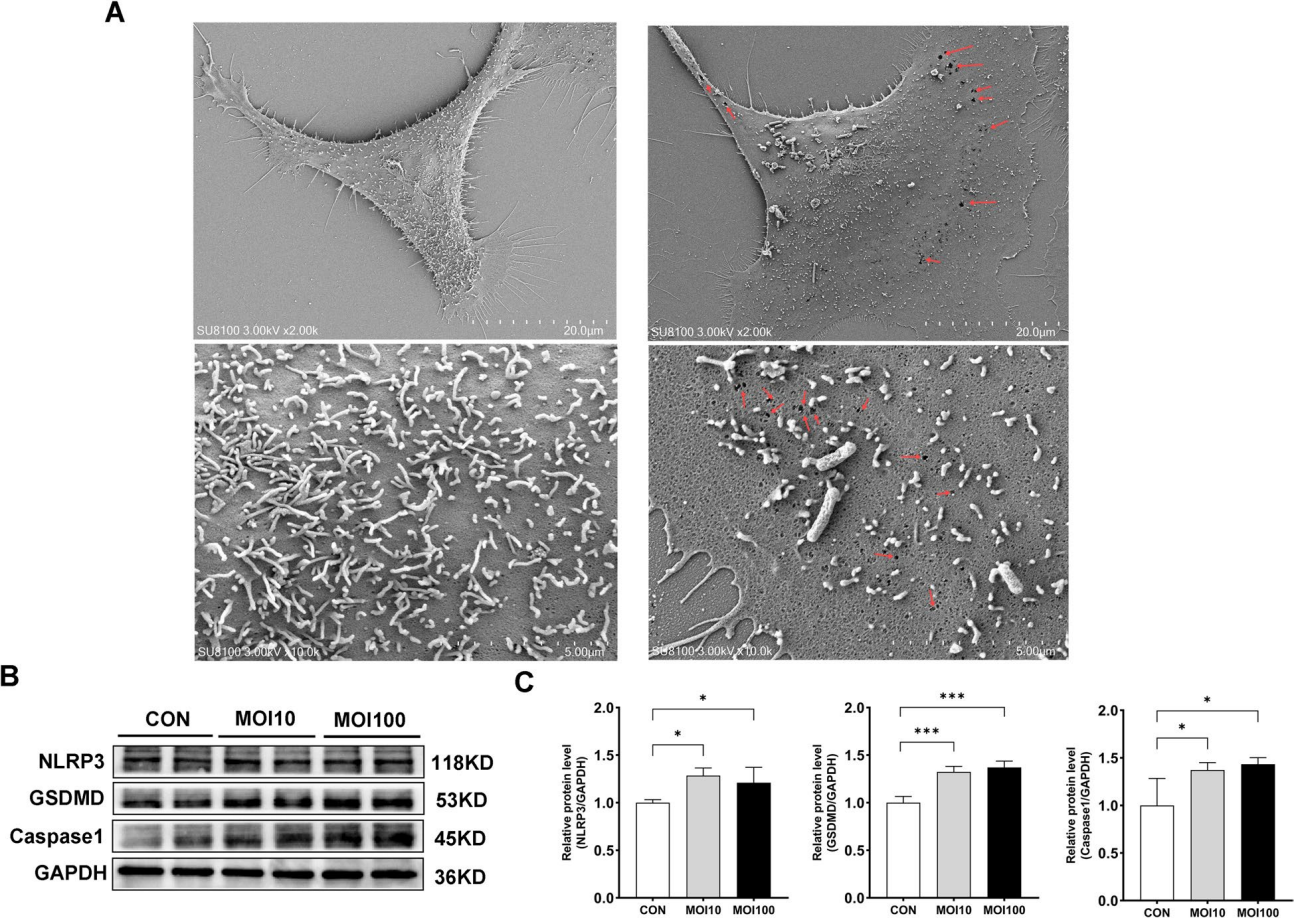
***G. parasuis*** **infection induces pyroptosis in 3D4/21 cells.** **A** *G. parasuis* induces pyroptotic morphological changes. Representative SEM images show the CON group (left panels) and the GPS group (right panels) at magnifications of ×2.0 k and ×10.0 k. Significant membrane disruption and pore formation are observed in the GPS group (red arrows), whereas the CON group maintains intact cellular membranes. **B** Western blotting analysis of NLRP3, Caspase1 and GSDMD expression in 3D4/21 cells after infection. **C** Quantification of protein expression levels using ImageJ. Data are presented as mean ± SD (*n* = 4). Significance relative to the control group: *p < 0.05, **p < 0.01, ***p < 0.001. CON indicates the uninfected control group, and GPS indicates the *G. parasuis*-infected group.

To assess the impact of *G. parasuis* infection on pyroptosis, 3D4/21 cells were infected at MOIs of 10 and 100 for 24 h. SEM analysis revealed significant membrane disruption and pore formation in the GPS group, which are hallmark morphological features of pyroptosis. In contrast, cells in the control group exhibited an intact morphology with abundant microvilli on their surface, without noticeable damage or pore formation (Figure 3A). Western blot analysis further revealed significantly increased levels of NLRP3, GSDMD, and Caspase-1, indicating that *G. parasuis* infection induces considerable pyroptosis in 3D4/21 cells (*p* < 0.05; Figures 3B and C).

### Analysis of ER stress signalling pathways induced by *G. parasuis* infection in 3D4/21 cells

**Figure 4:**
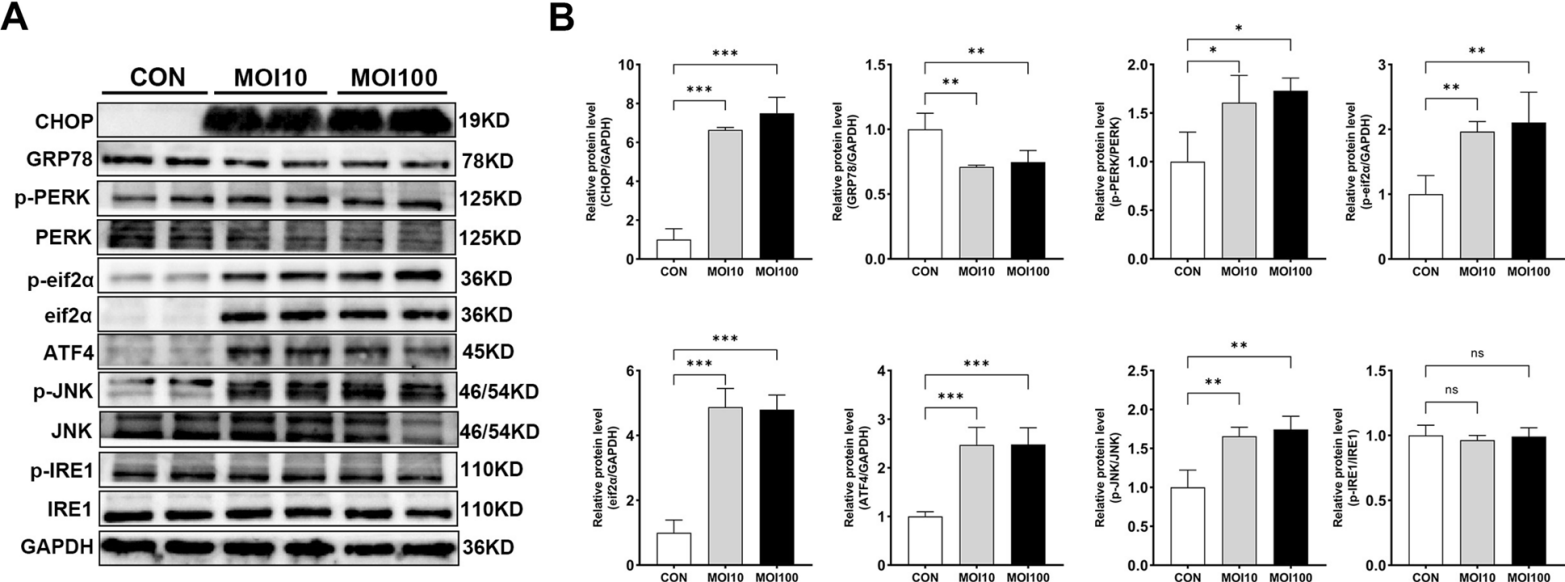
**Effects of** ***G. parasuis*** **infection on ER stress-related proteins in 3D4/21 cells**. **A** Western blotting analysis of CHOP, GRP78, p-PERK, PERK, p-eIF2α, eIF2α, ATF4, p-JNK, JNK, p-IRE1 and IRE1 expression in 3D4/21 cells after infection. **B** Quantification of protein expression levels using ImageJ. Uninfected cells served as the control group (CON). Data are presented as mean ± SD (*n* = 4). Significance relative to the control group: ns, not significant, *p < 0.05, **p < 0.01, ***p < 0.001.

To investigate whether *G. parasuis* infection activates ER stress in 3D4/21 cells, we analysed the expression levels of ER stress-related proteins by western blot. The proteins examined included CHOP, GRP78, p-PERK, PERK, p-eIF2α, eIF2α, ATF4, p-JNK, JNK, p-IRE1, and IRE1. As shown in Figure 4, infection with *G. parasuis* at MOIs of 10 and 100 led to a significant upregulation of CHOP, ATF4, p-PERK, p-eIF2α, eIF2α, and p-JNK levels compared to the control group (*p* < 0.05). Conversely, GRP78 expression was significantly decreased in the infected cells (*p* < 0.05). No significant changes were observed in the p-IRE1/IRE1 ratio.

**Figure 5.**
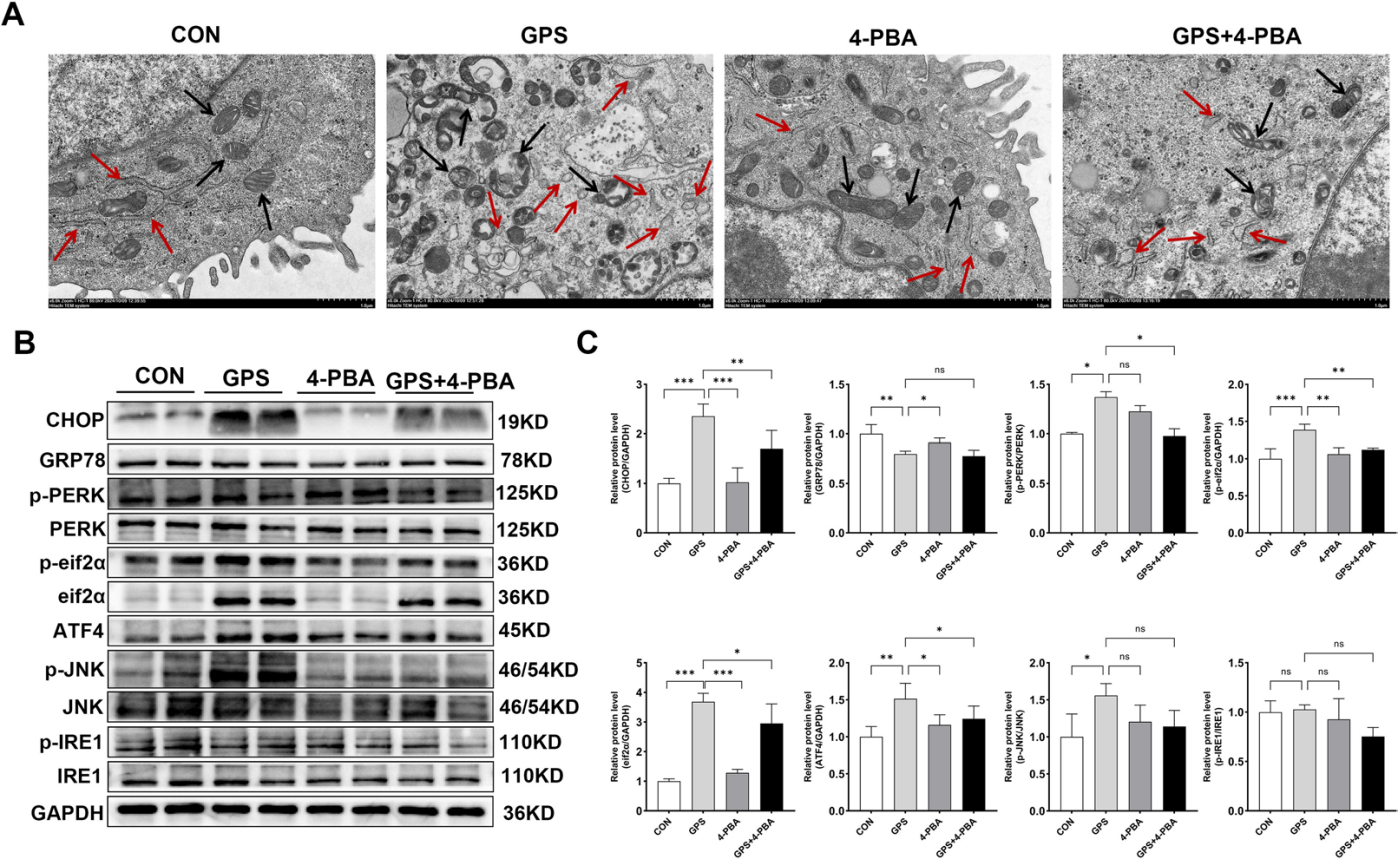
**4-PBA attenuates** ***G. parasuis*-induced ER stress in 3D4/21 cells.** **A** Representative TEM micrographs of 3D4/21 cells under different treatment conditions (magnification: × 6.0 k). The scale bar represents 1.0 μm. The endoplasmic reticulum (red arrows) and mitochondria (black arrows) are indicated. **B** Western blot analysis of CHOP, GRP78, p-PERK, PERK, eIF2α, p-eIF2α, ATF4, JNK, p-JNK, p-IRE1, and IRE1 protein expression levels in 3D4/21 cells under various treatments. **C** Quantitative analysis of protein expression levels was performed using ImageJ software. Data are presented as the mean ± SD (*n* = 4). Statistical significance is indicated as: ns, not significant, *p < 0.05, **p < 0.01, and ***p < 0.001. The four groups were: CON (uninfected control), GPS (*G. parasuis*, MOI = 10), 4-PBA (5 mM 4-PBA), and GPS + 4-PBA (pre-treated with 5 mM 4-PBA for 2 h before infection).

To further confirm the involvement of ER stress, we pre-treated 3D4/21 cells with 5 mM 4-PBA, an ER stress inhibitor, for 2 h prior to infection with *G. parasuis* (MOI = 10) for 24 h. TEM analysis revealed that *G. parasuis* infection induced typical ER stress-associated ultrastructural changes, including ER dilation, ribosomal detachment, and mitochondrial damage characterised by disrupted cristae and widened intracristal spaces (Figure 5A). Notably, these changes were substantially alleviated by 4-PBA pre-treatment.

Furthermore, 4-PBA markedly attenuated the *G. parasuis*-induced upregulation of CHOP, p-PERK, p-eIF2α, and ATF4 protein levels (*p* < 0.05; Figures 5B and C). Taken together, these findings indicate that *G. parasuis* infection activates ER stress in 3D4/21 cells via the PERK/eIF2α/ATF4 signalling pathway.

### Effect of GSK2656157 on *G. parasuis*-induced apoptosis and pyroptosis in 3D4/21 cells

**Figure 6:**
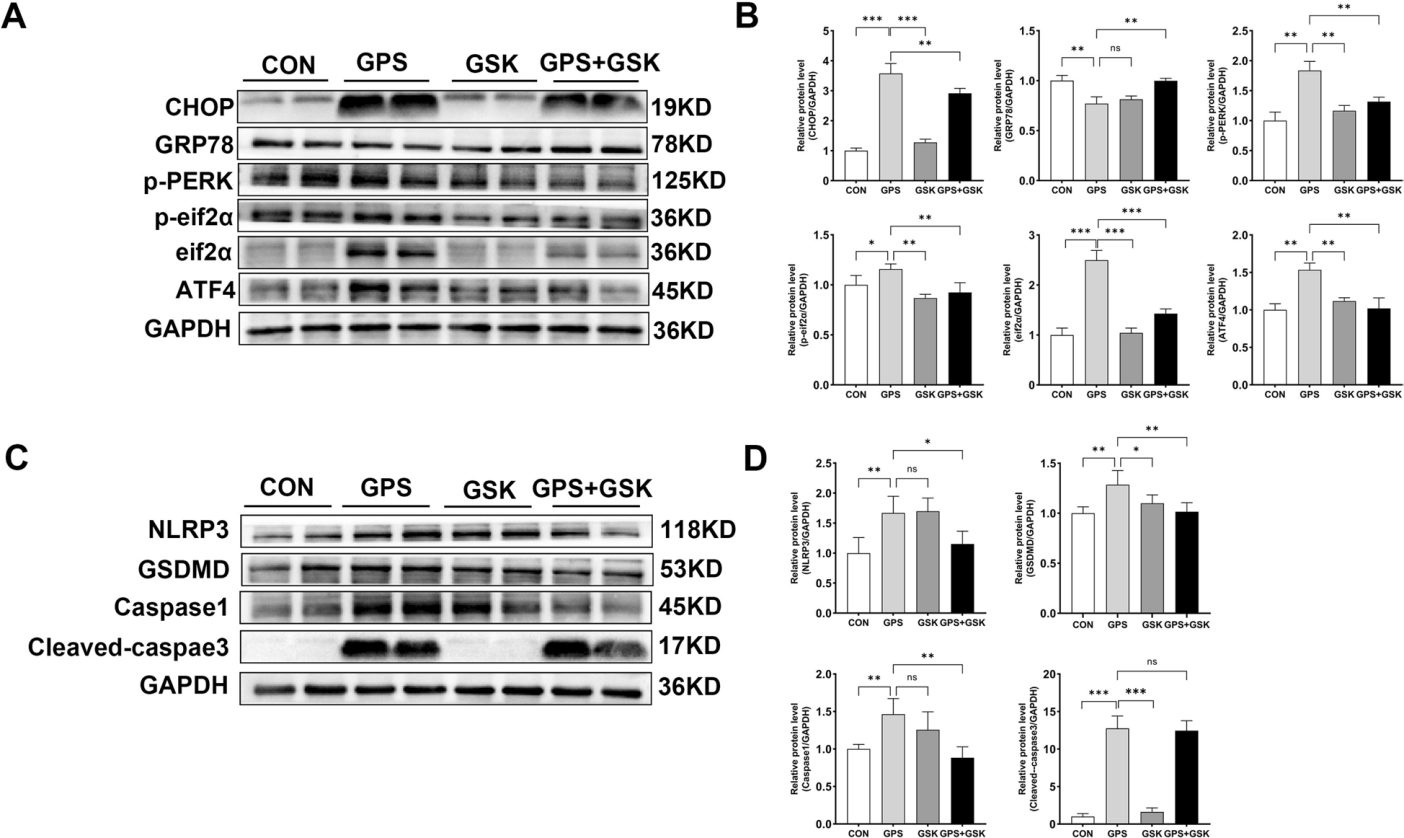
**Effects of GSK2656157 on PERK pathway activation, apoptosis, and pyroptosis-related protein expression induced by** ***G. parasuis***. **A** Western blot analysis of PERK pathway-related proteins (CHOP, GRP78, p-PERK, p-eIF2α, eIF2α, and ATF4) in CON (uninfected control), GPS (*G. parasuis*, MOI = 10), GSK (10 μM GSK2656157), and GPS + GSK (pre-treated with 10 μM GSK2656157 for 2 h before infection) groups. **B** Protein expression levels were quantified using ImageJ software. **C** Western blot analysis of pyroptosis-related proteins (NLRP3, GSDMD, Caspase-1) and the apoptosis-related protein (Cleaved Caspase-3) was conducted in the same four groups. **D** Quantitative analysis of these protein levels was also performed using ImageJ software. Data are presented as the mean ± SD (*n* = 4). Statistical significance is indicated as: ns, not significant, *p < 0.05, **p < 0.01, and ***p < 0.001.

To clarify the role of the PERK/eIF2α/ATF4 signalling pathway, 3D4/21 cells were pre-treated with the PERK inhibitor GSK2656157 (10 μM) for 2 h before *G. parasuis* infection (MOI = 10) for 24 h. Western blot analysis showed that GSK2656157 significantly suppressed the expression of CHOP, p-PERK, p-eIF2α, and ATF4, while markedly up-regulating GRP78 levels compared to the infected group without inhibitor treatment (*p* < 0.05; Figs. 6A and B). These findings indicate that GSK2656157 effectively inhibits the activation of the PERK/eIF2α/ATF4 pathway in response to *G. parasuis* infection.

We next examined the effects of GSK2656157 on *G. parasuis*-induced cell death. Notably, GSK2656157 treatment significantly reduced the protein levels of pyroptosis-associated markers, including NLRP3, GSDMD, and Caspase-1, relative to the infected group (*p* < 0.05; Figures 6C and D). However, there was no significant change in cleaved caspase-3 expression between the GPS + GSK and GPS groups, suggesting that GSK2656157 primarily modulates pyroptosis rather than apoptosis under these conditions.

### Metabolomic analysis of 3D4/21 cells following *G. parasuis* infection

**Figure 7:**
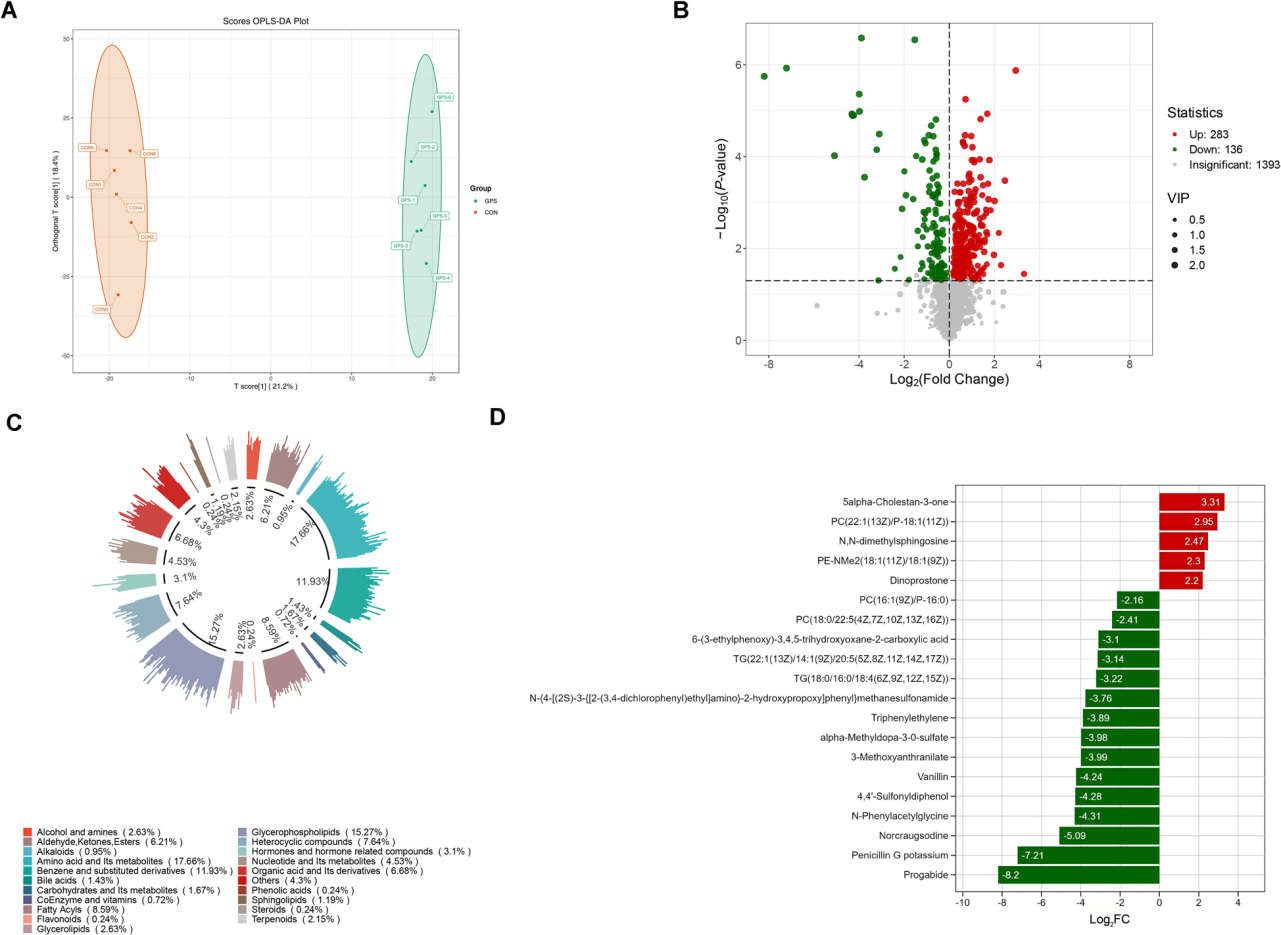
**Metabolic profiling of** ***G. parasuis*-infected 3D4/21 cells.A** OPLS-DA scores plot showing distinct metabolic profiles between the control (CON, uninfected) and *G. parasuis*-infected (GPS, MOI = 10) groups. **B** Volcano plot: Green points (downregulated), Red points (up-regulated), and grey points (non-significant) represent metabolites. X-axis: log_2_FC; Y-axis: -log_10_ *p*-value; point size: VIP value. **C** Circle chart of the proportion of primary classification of DEMs. **D** Top 20 most significant DEMs following *G. parasuis* infection. Up-regulated metabolites are shown in red, and down-regulated metabolites are shown in green.

To explore the metabolic alterations induced by *G. parasuis* infection, we performed a UPLC–MS/MS-based metabolomic analysis of 3D4/21 cells. As shown in the scores plot (Figure 7A), a clear separation was observed between the *G. parasuis*-infected and control groups, indicating distinct metabolic profiles and significant perturbations in response to infection.

A total of 419 differentially expressed metabolites (DEMs) were identified, with 283 up-regulated and 136 down-regulated (Figure 7B; Additional file 3). These DEMs were classified into 21 primary (Class I) metabolite categories (Figure 7C). The most prominent categories included amino acids and their metabolites (17.66%), glycerophospholipids (15.27%), and benzene and substituted derivatives (11.93%), suggesting that *G. parasuis* infection profoundly impacts amino acid metabolism and lipid remodelling.

To further characterise specific metabolic changes, the top 20 most significantly altered DEMs were visualised (Figure 7D). Among these, five metabolites were markedly up-regulated: 5α-Cholestan-3-one, PC(22:1(13Z)/P-18:1(11Z)), N,N-Dimethylsphingosine, PE-NMe2(18:1(11Z)/18:1(9Z)), and Dinoprostone. In contrast, fifteen metabolites were markedly down-regulated, including Progabide, Penicillin G potassium, Norcraugsodine, N-Phenylacetylglycine, 4,4'-Sulfonyl diphenol, vanillin, 3-methoxyanthranilate, α-methyldopa-3-O-sulfate, triphenylethylene, and several complex lipids such as PC(16:1(9Z)/P-16:0), PC(18:0/22:5), and triacylglycerol (TG) species.

**Figure 8:**
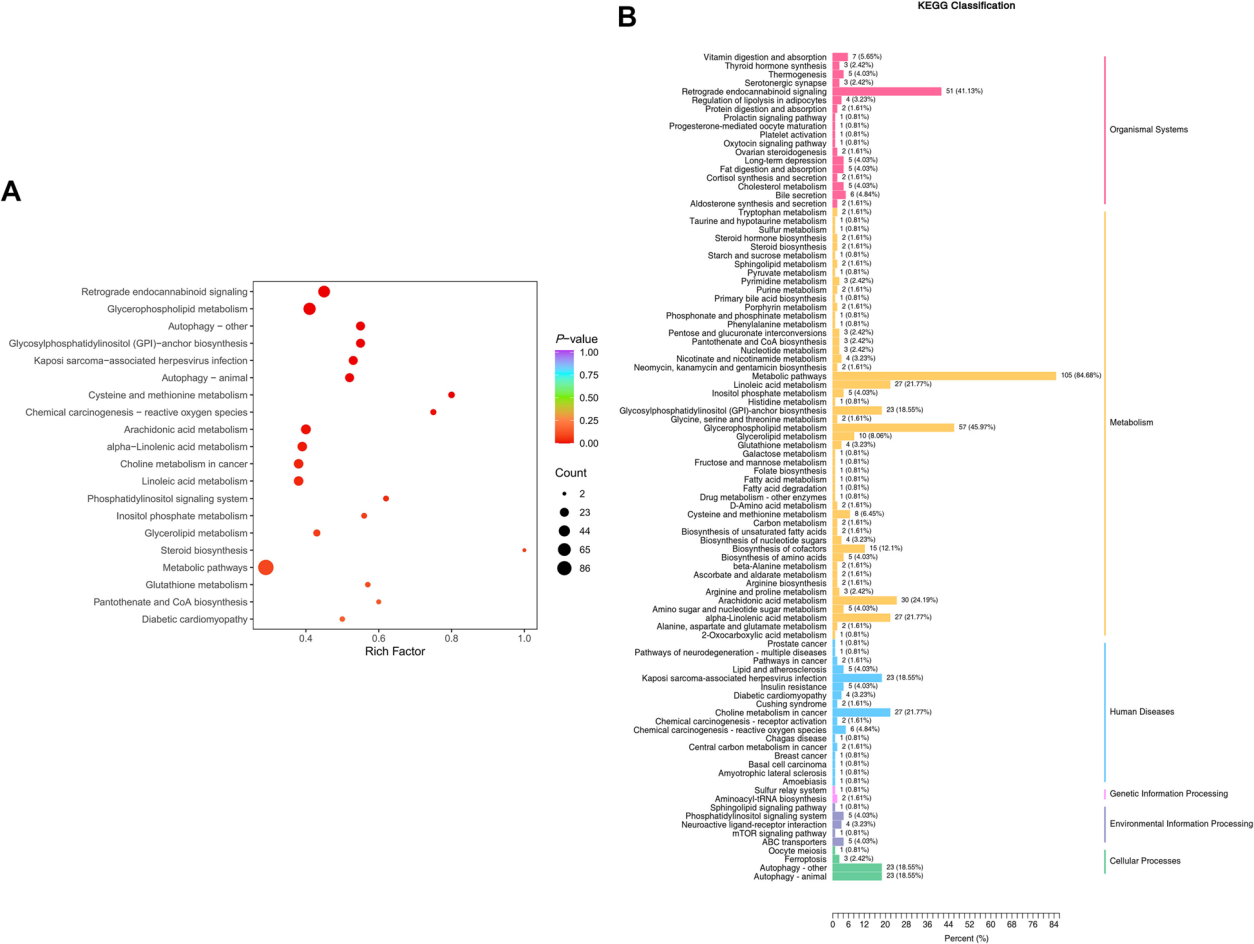
**KEGG enrichment and classification analysis of differential metabolites.** **A** KEGG enrichment analysis. The size and colour of the bubbles indicate the number of different metabolites and the degree of enrichment, respectively. **B** KEGG classification of the DEMs. The X-axis shows the proportion and number of metabolites annotated to each pathway, while the Y-axis lists the pathway names.

To identify key metabolic pathways altered in 3D4/21 cells upon *G. parasuis* infection, KEGG enrichment analysis was performed. As shown in Figure 8, pathway enrichment analysis revealed significant involvement of multiple pathways related to microbial infection and immune responses. These include retrograde endocannabinoid signalling, glycerophospholipid metabolism, autophagy, glycosylphosphatidylinositol (GPI)-anchor biosynthesis, cysteine and methionine metabolism, arachidonic acid metabolism, alpha-linolenic acid metabolism, linoleic acid metabolism, phosphatidylinositol signalling system, inositol phosphate metabolism, glycerolipid metabolism, glutathione metabolism, and pantothenate and CoA biosynthesis. Table 1 highlights the KEGG pathways associated with the top 10 up-regulated and down-regulated DEMs. Notably, metabolites PC(22:1(13Z)/P-18:1(11Z)) and PE-NMe2(18:1(11Z)/18:1(9Z)) were involved in several of these pathways.

**Table 1:** **KEGG pathways associated with the Top 10 up-regulated and down-regulated differential metabolites**

Index	Compounds	Class I	Formula	Type	KEGG pathways
MW0017077	5alpha-Cholestan-3-one	Hormones and hormone-related compounds	C27H46O	Up	–
MW0057507	PC(22:1(13Z)/P-18:1(11Z))	Glycerophospholipids	C48H92NO7P	Up	Glycerophospholipid metabolism, Arachidonic acid metabolism, Linoleic acid metabolism, alpha-Linolenic acid metabolism, Metabolic pathways, Retrograde endocannabinoid signalling, Choline metabolism in cancer
MW0008588	N,N-dimethylsphingosine	Sphingolipids	C20H41NO2	Up	–
MW0060337	PE-NMe2(18:1(11Z)/18:1(9Z))	Glycerophospholipids	C43H82NO8P	Up	Glycosylphosphatidylinositol (GPI)-anchor biosynthesis, Glycerophospholipid metabolism, Metabolic pathways, Autophagy, Retrograde endocannabinoid signalling, Kaposi sarcoma-associated herpesvirus infection
FDATN00416	Dinoprostone	Hormones and hormone-related compounds	C20H32O5	Up	Steroid biosynthesis, Metabolic pathways
MEDN0758	( ±)4-HDHA	Fatty Acyls	C22H32O3	Up	–
MEDP1528	Carnitine C18:1-OH	Fatty Acyls	C25H47NO5	Up	–
MEDP1391	Carnitine C18:2	Fatty Acyls	C25H45NO4	Up	–
MW0063221	Psychosine	Sphingolipids	C24H47NO7	Up	Sphingolipid metabolism
MW0170003	Veratramine	Benzene and substituted derivatives	C27H39NO2	Up	–
MW0007611	N-{4-[(2S)-3-{[2-(3,4-dichlorophenyl)ethyl]amino}-2-hydroxypropoxy]phenyl}methanesulfonamide	Benzene and substituted derivatives	C18H22Cl2N2O4S	Down	–
MW0158070	Triphenylethylene	Heterocyclic compounds	C20H16	Down	–
MW0103866	alpha-Methyldopa-3-0-sulfate	Organic acid and its derivatives	C10H13NO7S	Down	–
MEDL02283	3-Methoxyanthranilate	Benzene and substituted derivatives	C8H9NO3	Down	Tryptophan metabolism
MW0168600	Vanillin	Benzene and substituted derivatives	C8H8O3	Down	Metabolic pathways
MW0004457	4,4'-Sulfonyldiphenol	Benzene and substituted derivatives	C12H10O4S	Down	–
MEDN0061	N-Phenylacetylglycine	Amino acid and its metabolites	C10H11NO3	Down	Phenylalanine metabolism
MW0160208	Norcraugsodine	Benzene and substituted derivatives	C15H15NO3	Down	–
MW0109054	Penicillin G potassium	Others	C16H17KN2O4S	Down	–
MW0009528	Progabide	Amino acid and its metabolites	C17H16ClFN2O2	Down	–

## Discussion

*G. parasuis* initially colonises the upper respiratory tract and subsequently reaches the lungs, where it encounters PAMs, the first line of innate defence. Virulent strains can evade phagocytosis by PAMs, breach the pulmonary barrier, and cause systemic infection, leading to Glässer’s disease [24, 25]. In our study, infection of 3D4/21 cells (a PAM-derived cell line) with *G. parasuis* at MOIs of 10 and 100 induced a robust inflammatory response, evidenced by the upregulation of IL-6, IL-8, IL-1β, and TNF-α, accompanied by reduced cell viability, indicating both immune activation and cellular injury.

To further investigate the mechanisms underlying this cellular damage, we examined the involvement of programmed cell death pathways. Our results demonstrated that *G. parasuis* infection induced apoptosis in 3D4/21 cells, as indicated by increased expression of Bax and cleaved caspase-3, and decreased Bcl-2 levels, thereby shifting the balance towards apoptosis. Bcl-2 and Bax regulate mitochondrial apoptosis by forming hetero- or homodimers: Bcl-2 inhibits apoptosis, whereas Bax promotes it by enhancing mitochondrial membrane permeabilisation and activating caspase-3 [26, 27]. Flow cytometry with Annexin V/PI staining further confirmed a significant increase in apoptosis in *G. parasuis*-infected cells compared to controls.

In addition to apoptosis, *G. parasuis* induced pyroptotic features in 3D4/21 cells, including membrane swelling and rupture, as observed by SEM. Pyroptosis, characterised by GSDMD-mediated pore formation, is triggered via NLRP3 inflammasome–dependent caspase-1 activation [28, 29]. Consistently, *G. parasuis* infection significantly up-regulated NLRP3, GSDMD, and caspase-1 expression at 24 h post-infection (hpi), indicating activation of the canonical pyroptotic pathway in PAMs.

ER stress plays a critical role in pathogen-induced diseases [13]. GRP78, a central component of the UPR, acts as an ER chaperone that binds to misfolded proteins to facilitate proper folding. However, under ER stress, it dissociates from IRE1, PERK, and ATF6, thereby activating these UPR pathways and their downstream effects [30]. In our study, *G. parasuis* infection resulted in a significant reduction in GRP78 protein levels in 3D4/21 cells, indicating degradation of GRP78 and subsequent initiation of ER stress.

Among the UPR branches, PERK and IRE1 are key mediators of stress-induced cell death [31, 32]. Therefore, we focused on their activation following *G. parasuis* infection. Notably, expression of CHOP, p-PERK, p-eIF2α, ATF4, and p-JNK was markedly increased, whereas IRE1 signalling remained unaffected. PERK activation leads to eIF2α phosphorylation, which suppresses global protein synthesis while selectively enhancing translation of ATF4 and CHOP, both of which mediate apoptosis and inflammation [33].

A well-known ER stress inhibitor, 4-PBA, has been commonly used in previous cellular studies at concentrations of 5 mM to effectively alleviate ER stress [34]. In our study, TEM revealed classical ER stress features in infected cells, including ER dilation, ribosome detachment, and mitochondrial damage. These pathological changes were reversed by 5 mM 4-PBA, which also suppressed *G. parasuis*-induced expression of CHOP, p-PERK, p-eIF2α, and ATF4, confirming the involvement of the PERK pathway in the ER stress response.

To probe the functional consequences of PERK activation, we used GSK2656157, a selective PERK inhibitor. GSK2656157 (10 μM) significantly suppressed PERK pathway activation and down-regulated the expression of NLRP3, GSDMD, and Caspase-1, indicating that *G. parasuis* triggers pyroptosis in 3D4/21 cells via the PERK/eIF2α/ATF4/CHOP axis. This outcome is consistent with findings by Ma et al., who reported that PERK inhibition alleviates *Bacillus Calmette-Guérin*-induced pyroptosis in macrophages [35]. Recent research suggests that thioredoxin-interacting protein (TXNIP) mediates ER stress-induced activation of the NLRP3 inflammasome, with elevated CHOP expression promoting TXNIP overexpression and subsequent inflammasome activation [36]. Therefore, we speculate that *G. parasuis* may induce pyroptosis through a PERK–CHOP–TXNIP/NLRP3 pathway. Interestingly, while GSK2656157 inhibited pyroptosis markers, it did not reduce apoptosis in infected 3D4/21 cells, suggesting that *G. parasuis* induces apoptosis via a PERK-independent mechanism.

To further investigate the cellular response to *G. parasuis* infection, we performed untargeted metabolomics analysis using UPLC-MS/MS in 3D4/21 cells. This approach identified 283 up-regulated and 136 down-regulated metabolites. KEGG pathway enrichment analysis revealed several metabolic pathways associated with microbial infection and immune response.

Among the altered pathways, retrograde endocannabinoid signalling, initially known for maintaining nervous system homeostasis [37], has recently been implicated in regulating immune responses during bacterial sepsis [38]. Glycerophospholipid metabolism was also disrupted, reflecting changes in membrane structure and transmembrane signalling; such disruptions are associated with inflammation and oxidative stress during infection [39–41].

Alterations in glutathione metabolism further suggested increased oxidative stress and immune activation. Glutathione is a key antioxidant that protects against ROS and microbial invasion [42]. Notably, several pathogens have been shown to manipulate host glutathione metabolism: *Streptococcus pyogenes* utilises host glutathione for survival and immune evasion [43], and *Borrelia burgdorferi* modulates this pathway to affect cytokine production [44].

We also identified changes in cysteine and methionine metabolism, which are central to sulfur amino acid pathways. Cysteine serves as a precursor to glutathione, contributing to redox homeostasis, while methionine plays roles in protein synthesis, epigenetic regulation, polyamine synthesis, and maintaining membrane phospholipid balance [45–47].

Arachidonic acid metabolism was another significantly enriched pathway. It generates eicosanoids–bioactive lipids that regulate inflammation and immunity [48, 49]. Additionally, perturbations in pantothenate and CoA biosynthesis were observed. As a precursor to CoA, pantothenate supports fatty acid metabolism and the tricarboxylic acid (TCA) cycle [50]. CoA is crucial for immune cell energy metabolism and can modulate their proliferation, differentiation, and effector functions [51].

Together, these findings indicate that *G. parasuis* infection triggers broad metabolic reprogramming in PAMs. The altered pathways, many of which are intimately involved in inflammation and host defence, underscore the profound impact of infection on host cell metabolism. Future studies are needed to elucidate the specific functional consequences of these metabolic shifts during the course of infection.

This study reveals that *G. parasuis* infection induces strong inflammatory responses and cell death in PAMs, characterised by activation of both apoptosis and pyroptosis. We identified the PERK/eIF2α/ATF4/CHOP axis as a key mediator of ER stress and pyroptosis, while apoptosis occurred via a PERK-independent pathway. Metabolomics analysis further demonstrated significant disruptions in glutathione, glycerophospholipid, and arachidonic acid metabolism. Together, these findings highlight the complex interplay between ER stress, cell death, and metabolic dysregulation during *G. parasuis* infection. These findings offer new insights into the mechanisms of *G. parasuis* pathogenesis and may guide future studies to elucidate the regulatory interplay among ER stress, programmed cell death, and metabolic reprogramming, and to identify novel therapeutic targets.

## Supplementary Information

**Additional file 1:** **Sequences of primers used for RT-qPCR.**

**Additional file 2:** **The metabolite identification analytical conditions.**

**Additional file 3:** **Significant differential metabolites identified following G. parasuis infection.**

## Data Availability

The datasets used and/or analysed during the current study are available from the corresponding author on reasonable request.
