# Supplementary material for: Glaesserella parasuis infection triggers endoplasmic reticulum stress-mediated pyroptosis via PERK/eIF2α/ATF4 axis and metabolic reprogramming in porcine alveolar macrophages
Source: Vet Res. 2025 Jul 15;56:150. doi: 10.1186/s13567-025-01580-2 (PMC12265192; doi:10.1186/s13567-025-01580-2)
Supplement: Supplementary file 1 — Additional file 1: Sequences of primers used for RT-qPCR. [file 13567_2025_1580_MOESM1_ESM.docx]

**Additional file 1 Sequences of Primers Used for RT-qPCR**

| Primer | Sequence |
| --- | --- |
| *IL-6-F* | CCAGGAACCCAGCTATGAAC |
| *IL-6-R* | CTGCACAGCCTCGACATT |
| *IL-8-F* | TCTTGGCAGTTTTCCTGCTTT |
| *IL-8-R* | AATTTGGGGTGGAAAGGTGT |
| *IL-1β-F* | GTGGCAGGACCTACACTCTTC |
| *IL-1β-R* | TTCCTTCAGAATGCCGTCCTC |
| *TNF-α-F* | GGAGTAGATGAGGTACAG |
| *TNF-α-R* | GACTCAGATCATCGTCTC |
| *GAPDH-F* | TCGGAGTGAACGGATTTG |
| *GAPDH-R* | CCTGGAAGATGGTGATGG |
