# Supplementary material for: Glaesserella parasuis infection triggers endoplasmic reticulum stress-mediated pyroptosis via PERK/eIF2α/ATF4 axis and metabolic reprogramming in porcine alveolar macrophages
Source: Vet Res. 2025 Jul 15;56:150. doi: 10.1186/s13567-025-01580-2 (PMC12265192; doi:10.1186/s13567-025-01580-2)
Supplement: Supplementary file 2 — Additional file 2: The metabolite identification analytical conditions. [file 13567_2025_1580_MOESM2_ESM.docx]

**Additional file 2: The Metabolite Identification Analytical Conditions**

UPLC Conditions: Samples were analyzed using two LC/MS methods. For positive ion mode, separation was performed on a Waters ACQUITY Premier HSS T3 Column (1.8 µm, 2.1 mm × 100 mm) with a mobile phase consisting of 0.1% formic acid in water (solvent A) and 0.1% formic acid in acetonitrile (solvent B). The gradient elution was as follows: 5% to 20% B over 2 min, increased to 60% B in the next 3 min, raised to 99% B in 1 min and held for 1.5 min, then returned to 5% B in 0.1 min and held for 2.4 min. The column temperature was maintained at 40 °C, with a flow rate of 0.4 mL/min and an injection volume of 4 μL. The same gradient was applied for negative ion mode analysis.

MS Conditions (AB Sciex): Data acquisition was performed in information-dependent acquisition (IDA) mode using Analyst TF 1.7.1 Software. Source parameters were set as follows: ion source gas 1 (GAS1) and gas 2 (GAS2), 50 psi; curtain gas (CUR), 25 psi; temperature (TEM), 550 °C; declustering potential (DP), ±60 V for positive and negative modes, respectively; ion spray voltage floating (ISVF), ±5000 V or ±4000 V for positive and negative modes, respectively. TOF MS scan parameters included a mass range of 50–1000 Da, accumulation time of 200 ms, and dynamic background subtraction enabled. Product ion scan parameters were set to a mass range of 25–1000 Da, accumulation time of 40 ms, collision energy of ±30 V for positive and negative modes, collision energy spread of 15, unit resolution, charge state of 1, intensity threshold of 100 cps, exclusion of isotopes within 4 Da, mass tolerance of 50 ppm, and a maximum of 18 candidate ions monitored per cycle.
